# Effects of Rock Climbing Exercise on Physical Fitness among College Students: A Review Article and Meta-analysis

**Published:** 2018-10

**Authors:** Lun LI, An RU, Ting LIAO, Shisi ZOU, Xiao Hong NIU, Yong Tai WANG

**Affiliations:** 1. Dept. of Physical Education, China University of Geosciences, Wuhan, China; 2. National Training Office, Shandong Sport University, Shandong, China; 3. Dept. of Swimming Teaching and Research, Wuhan Sports University, Wuhan, China; 4. College of Nursing and Health Sciences, The University of Texas, Tyler, Texas, USA

**Keywords:** Physical fitness, Function, Rock climbing, College students

## Abstract

**Background:**

The aim of this study was to systematically determine the effects of rock climbing on College Students’ physical fitness by means of Meta-analysis.

**Methods:**

Studies investigated the possible fitness benefits of rock climbing were identified through a computerized search of six electronic databases: China National Knowledge Infrastructure, China Science Periodical Database, PubMed, Web of Science, SPORTDiscus and PsycINFO. Effects of rock climbing exercise intervention trials ≥4 wk, published in English and Chinese between Jan 1996 and May 2016, including between subject and within subject designs, were reviewed. Nine studies were included in this meta-analysis. Ten selected variables in this meta-analysis were: Body fat percentage, VO_2_max, Heart rate, Handgrip strength, Lower limb pedaling power, Vertical Jump, Push-Ups, Pull-Ups, Sit-Ups, and Sit-and-reach. The effect sizes of these ten variables were calculated (*P*<0.05) and forest plots along with effective sizes were presented.

**Results:**

Rock climbing can significantly improve Handgrip strength, Lower limb pedaling power, Vertical Jump, Push-Ups, Pull-Ups, Sit-Ups and Sit-and-reach (*P*<0.01), and significantly increase VO_2_max (*P*<0.05), however, rock climbing did not show significant improvement on Heart rate and Body fat percentage.

**Conclusion:**

As a newly popular physical exercise, rock climbing has a significantly positive impact on the physical fitness among college students. Rock climbing may be more effective if the college students engage in it for a longer term.

## Introduction

Nowadays, the pace of modern life has been rapidly accelerated, and sedentary lifestyle plus lack of physical activity is becoming a growing health problem (1), especially among college students. Due to the lack of interest in physical exercise, most college students in China may spend little time on the exercise and could not form good exercise habits. Therefore, their physical fitness and health condition are of concern. Meanwhile, there are other factors such as the lack of attractive fitness programs and lack of fitness facilities at the Chinese Universities may lead to the potential decline of the physical fitness among college students (2).

Rock climbing has been recognized for many years as a recreational physical activity or entertainment that links its peculiar way of sport with the beauty of nature mountain environment (3). Rock climbing as a physical education course may attract the college students to participate in physical activities because current competitive sport events may not meet the needs or interests of these college students (4). Rock climbing is not only a leisure physical activity but also becomes competitive sport (5). In Brazil, Rio De Janeiro, on the International Olympic Committee (IOC) 129th plenary session, IOC officially announced that competitive climbing was accepted as an official game in 2020 Tokyo Olympic (6). Therefore, it would require us to have new understandings and proper development plans for this competitive rock climbing sport.

The physical benefits from rock climbing have been investigated in numerous studies (7–11). In rock climbing, the muscular system and facet joints can get plenty of exercises so that it should have a good effect on the balanced development of various parts of the body (7). During rock climbing, the climbers face variety of different angles and heights of the rock wall and have to overcome her/his body-weight and continuously complete turnarounds, pull-ups, even adventure actions like jumping to different spots by using the artificial handholds on the rock wall. Rock climbing has high and comprehensive physical demands on the climbers, such as the requirements on the muscle power and endurance of upper and lower limbs, hands and feet, waist and abdominal, and the truck and hip flexibilities (8, 11). Muehlbauer and associate examined the effect of an indoor climbing training program on core/handgrip strength and trunk mobility in men and women and concluded that maximal isometric strength of trunk flexors/extensors, trunk mobility (flexibility) in sagittal and front planes, and handgrip strength were significantly improved after 8 wk of indoor climbing training (8). A principal component analysis was conducted to identify important physical and physiological factors in climbing and climbing training factors are strength, anaerobic power and percentage body fat (11). Younger climbers can almost match an adult climber’s VO_2_peak without any additional training, and that aerobic potential is not a limiting factor to climbing performance (9, 10). However, the systematical reviews and meta-analyses focusing on the effects of rock climbing on physical fitness among college students have not been well documented.

Therefore, the purpose of this study was to systematically determine the effects of rock climbing on College Students’ physical fitness by means of Meta-analysis. We hope that the results of this study can provide useful information for creating and teaching rock climbing course in universities.

## Methods

### Search strategy

Studies that investigated the possible physical fitness benefits of rock climbing were identified through a computerized search of six electronic databases: China National Knowledge Infrastructure (CNKI), China Science Periodical Database (CSPD), PubMed, Web of Science, SPORTDiscus and PsycINFO. Articles published from Jan 1st, 1996 to May 1st, 2016 were included in this study. Eligible studies were included if they met all of the following criteria: 1) the effects of rock climbing interventions were examined; 2) research subjects were college students or age between 18 to 38 yr old; 3) the studies had the consistent hypotheses and experimental methods; 4) studies had original and complete data; 5) the test venue was the artificial rock climbing wall; 6) articles were written in English or Chinese; 7) all data required for meta-analysis included the means, standard deviations (SD) and sample sizes from the pre-test and post-test. Studies were excluded if 1) study was repeatedly published by the same researcher(s); 2) study had incomplete data.

### Study selection

The searches of the eligible studies in the electronic databases were performed in May 2016. After excluding duplications, 248 articles were retrieved. Nineteen studies were identified after evaluating titles and abstracts of the articles by two reviewers (LL, YTW). After carefully reviewing the full text of these articles, two studies were excluded due to the repeated publications; eight studies were excluded because the data of pre-test and post-test were incomplete. Based on final consensus of two reviewers, nine studies were included in this meta-analysis (12–20) with five studies in Chinese, four studies in English. Among these nine studies, four studies were between-subject design (12–15) and the remains were within-subject design (16–20). However, the data before and after the rock climbing intervention from experimental (intervention) group were used and treated as within-subject design study. The male and female participants’ data (14) were separately used as two sets of independent data into this meta-analysis. The core strength measured was treated as rock climbing specific strength variable in the present study (19, 20).

### Data extraction

All data were reviewed and separately extracted using a standardized form of Excel in Microsoft^®^ Office 2010 (Microsoft Corporation, Redmond, WA, USA). The following study characteristics were recorded if they were available: author, year of publication, type of study, frequency and duration of intervention studies. Moreover, all of the nine studies covering five aspects of physical fitness in terms of body composition; physiological function; muscle strength; muscle endurance; and body flexibility and a total of 10 variables consisting of Body fat percentage, VO_2_max, Heart rate, Handgrip strength, Lower limb pedaling power, Vertical Jump, Push-ups, Pull-ups, Sit-ups and Sit-and-reach, as shown in Table 1.

**Table 1:** Five aspects of physical fitness consisting of 10 Variables

***Aspects***	***Variables***
Body Composition	Body Fat Percentage
Body Function	Maximal Oxygen Uptake (VO_2_max), Heart Rate
Muscle Power	Handgrip Strength, Lower Limb Pedaling Power, Vertical Jump
Muscle Endurance	Push-ups, Pull-ups, Sit-ups
Body Flexibility	Sit-and-reach

These nine studies involved in 149 participants who were between the ages of 18–38 yr old and the intervention periods ranged from 4 to 24 wk. The basic information of each study in terms of the author(s), type of study, age (mean+SD), group (sample) size, intervention period, and variables is presented in Table 2.

**Table 2:** Nine included rock climbing studies for physical fitness

***Studies***	***Type of study***	***Age mean+SD***	***Group size***	***Intervention period***	***Variables***
Aras & Ewert (12)	Between Subject Design	21.11±2.3	9	8 wk	② ③
Li (13)	Between Subject Design	18–22 age range	20	8 wk	④ ⑤ ⑧
Gao (14)	Between Subject Design	20.75±0.8 Male	18	24 wk	①②③④⑤
		20.38±0.9 Female			⑥ ⑩
Jiang (15)	Between Subject Design	36.2±2.5	10	12 wk	③ ④ ⑤ ⑩
Cargo (16)	Within Subject Design	20.69±1.3	16	7 wk	① ④
Cuerdo & Pagaduan (17)	Within Subject Design	18.2 ± 1.8	37	4 wk	① ④ ⑥ ⑦ ⑧
Kasundra & Jethwa (18)	Within Subject Design	16 to 27	22	4 wk	⑧ ⑨
Shi (19)	Within Subject Design	18–22 age range	7	9 wk	④ ⑥ ⑦ ⑧ ⑨
Shi (20)	Within Subject Design	20±2.0	10	12 wk	① ④ ⑦ ⑨ ⑩

Abbreviations: ①Body Fat Percentage, ②VO_2_max, ③Heart Rate, ④Handgrip Strength, ⑤Lower Limb Pedaling Power, ⑥Vertical Jump, ⑦Push-Ups, ⑧Pull-Ups, ⑨Sit-Ups, ⑩Sit-and-reach

### Statistical analysis

In order for the data to be included in the meta-analysis, at least three sets of data from pre-test and post-test were required for effective size (ES) calculation (21).

The effective sizes of these ten variables were calculated and the forest plots were generated from each study (22). Positive values of ES mean favorable outcome for the intervention and negative values of ES mean non-favorable outcome for the intervention. Usually, ES is considered small (0.2 ≤ ES < 0.4), medium or moderate (0.4 ≤ ES<0.6) and large (ES≥0.6) with *P*<0.05 (23). The analyses of overall ES were implemented using random-effects model (24). The forest plots were generated from Excel Chart Function in Microsoft^®^ Office 2010 (Microsoft Corporation, Redmond, WA, USA) (21).

## Results

The results of the effect sizes and forest plots of the aforementioned ten variables are described as follows.

### Body fat percentage

Five sets of the Body Fat Percentages data from four studies involving 81 participants were utilized (14, 16, 17, 20) in the meta-analysis. Two independent sets of test data of male and female were included (14). Overall, there was no a significant difference between the pre-test and posttest in the Body Fat Percentage (95% CI, −1.22–0.43), and the mean ES is −0.83, *P*=2.0 in Table 3, considered a large ES, but is not statistically significant.

**Table 3:** Effective size (ES) and forest plot of body fat percentage

***Effect Size***					***Forest Plots***
***Studies***	***Weight***	***Random, 95%CI***			***Random, 95% CI***
		***ES***	***Low***	***High***	
Gao (14) (Male)	7.71%	−3.14	−4.52	−1.76	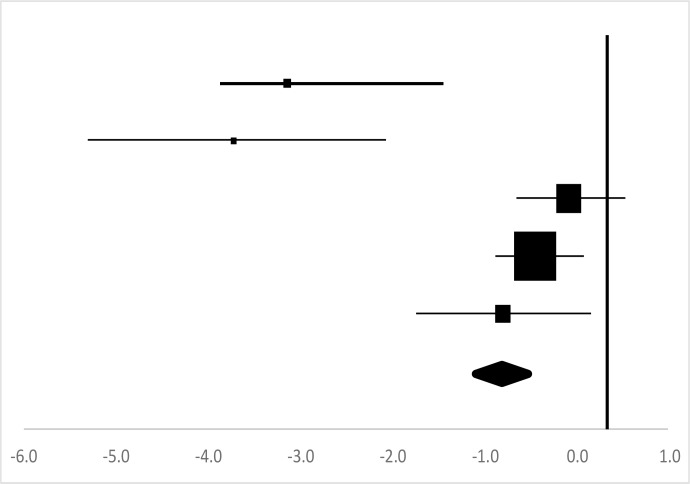
Gao (14) (Female)	6.38%	−3.72	−5.25	−2.19	
Cargo (16)	25.31%	−0.08	−0.77	0.61	
Cuerdo & Pagaduan (17)	44.35%	−0.44	−0.91	0.02	
Shi (20)	16.24%	−0.79	−1.70	0.12	
Total	100%	−0.83	−1.22	−0.43	Test for overall effect: Z=−4.07 (*P*=2.0)

Heterogeneity: Q=30.87, *df*=5, C=187.70, T^2^=1.14

### VO_2_max

Three sets of the VO_2_max data from two studies involving 27 participants were utilized in the meta-analysis (12, 14). Two independent data sets of male and female (14) were included. Overall, there was a significant difference between the pre-test and posttest in the VO_2_max (95% CI, 0.14 – 1.38), and the mean ES is 0.76, *P*<0.05 in Table 4, considered a large ES.

**Table 4:** Effective size (ES) and forest plot of VO_2_max

***Effect Size***					***Forest Plots***
***Studies***	***Weight***	***Random, 95%CI***			***Random, 95% CI***
		***ES***	***Low***	***High***	
Aras & Ewert (12)	37.64%	0.35	−0.58	1.28	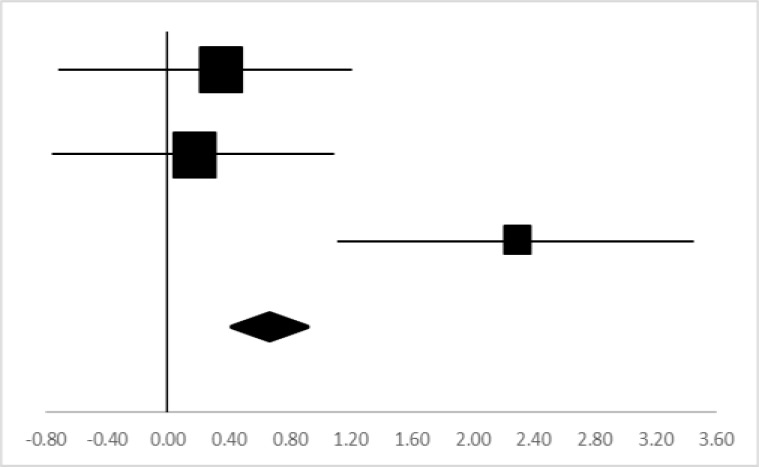
Gao (14) (Male)	38.01%	0.18	−0.75	1.11	
Gao (14) (Female)	24.35%	2.30	1.11	3.49	
Total	100%	0.76	0.14	1.38	Test for overall effect: Z=2.42 (*P*<0.05)

Heterogeneity: Q=8.67, *df*=2, C=8.35, T^2^=0.80

### Heart rate

Four sets of the Heart Rate data from three studies involving 37 participants were used in the meta-analysis (12, 14, 15). Overall, there was no significant difference between the pre-test and posttest in the Heart Rate (95%CI, −1.14–0.18), and the mean EF is −0.79, *P*=2.0 in Table 5, considered a large ES, but is not statistically significant.

**Table 5:** Effective size (ES) and forest plot of Heart Rate

***Effect Size***					***Forest Plots***
***Studies***	***Weight***	***Random, 95%CI***			***Random, 95% CI***
		***ES***	***Low***	***High***	
Gao (14) (Chinese Male)	15.1%	−3.67	−5.18	−2.15	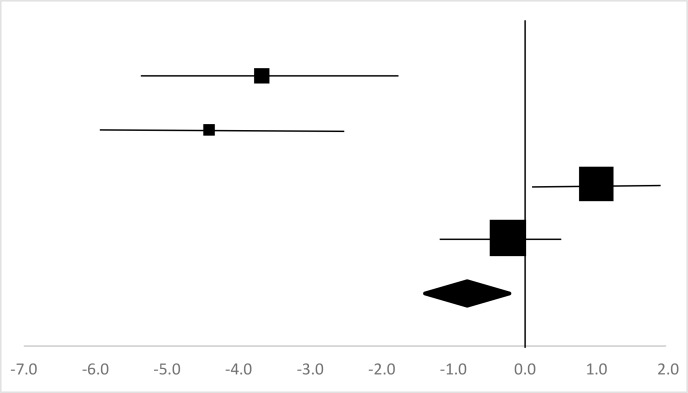
Gao (14) (Chinese Female)	11.97%	−4.4	−6.11	−2.69	
Jiang (15) (Chinese)	36.29%	1.02	0.09	1.95	
Aras & Ewert (12)	36.64%	−0.22	−1.14	0.71	
Total	100%	−0.79	−1.40	−0.18	Test for overall effect: Z=−2.56 (*P*=2.0)

Heterogeneity: Q=46.89, *df*=4, C=8.99, T^2^=4.88

### Handgrip strength

Eleven sets of the handgrip strength date from seven studies involving 118 participants were used in the meta-analysis (13–17, 19, 20). The male and female data (14), as well as the left hand and right-hand data (14, 15), were included. Overall, there was a significant difference the pre-test and post-test in Handgrip Strength (95% CI, 0.52–1.10), and the mean ES is 0.81, *P*<0.01 in Table 6, considered a large ES.

**Table 6:** Effective size (ES) and forest plot of Handgrip Strength

***Effect Size***					***Forest Plots***
***Studies***	***Weight***	***Random, 95%CI***			***Random, 95% CI***
		***ES***	***Low***	***High***	
Li (13)	8.22%	3.24	2.30	4.18	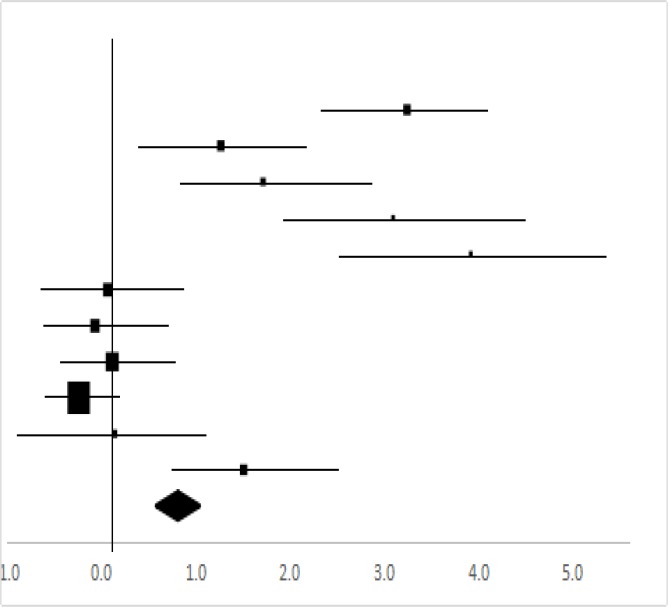
Gao (14) (Male, left)	7.26%	1.27	0.25	2.28	
Gao (14) (Male, right)	6.47%	1.71	0.63	2.80	
Gao (14) (Female, left)	4.2%	3.09	1.72	4.46	
Gao (14) (Female, right)	3.22%	0.28	2.34	5.50	
Jiang (15) (left)	9.3%	0.07	−0.81	0.94	
Jiang (15) (right)	9.3%	−0.07	−0.94	0.81	
Cargo (16)	13.59%	0.12	−0.58	0.81	
Cuerdo & Pagaduan (17)	24.08%	−0.24	−0.70	0.22	
Shi (19)	6.82%	0.14	−0.91	1.19	
Shi (20)	7.51%	1.51	0.52	2.50	
Total	100%	0.81	0.52	1.10	Test for overall effect: Z=5.43 (*P*<0.01)

Heterogeneity: Q=87.16, *df*=5, C=53.32, T^2^=1.45

### Lower Limb Pedaling Power

Four sets of Lower Limb Pedaling Power data from three studies involving 48 participants were used in the meta-analysis (13–15). Overall, there was significant difference between the pre-test and posttest in Lower Limb Pedaling Power (95%CI, 0.15–0.57), and the mean ES is 0.36, *P*≤0.01 in Table 7, considered a medium ES.

**Table 7:** Effective size (ES) and forest plot of Lower Limb Pedaling Power

***Effect Size***					***Forest Plots***
***Studies***	***Weight***	***Random, 95%CI***			***Random, 95% CI***
		***ES***	***Low***	***High***	
Li (13)	10.9%	6.67	5.08	8.26	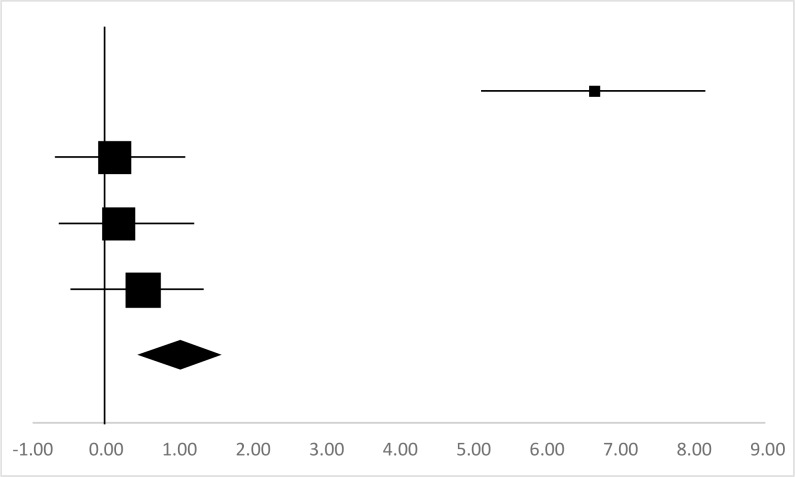
Gao (14) (Male)	29.08%	0.12	−0.8	1.05	
Gao (14) Female)	29.02%	0.17	−0.75	1.10	
Jiang (15)	31%	0.97	0.43	1.51	
Total	100%	0.36	0.15	0.57	Test for overall effect: Z=3.53 (*P*<0.01)

Heterogeneity: Q=57 *df*=4, C=12.03, T^2^=4.49

### Vertical Jump

Four sets of Vertical Jump data from three studies involving 62 participants were used in the meta-analysis (14, 17, 19). Overall, there was a significant difference between the pre-test and posttest in the Vertical Jump (95% CI, 0.29–1.18), and the mean ES is 0.73, *P*<0.01 in Table 8, considered a large ES.

**Table 8:** Effective size (ES) and forest plot of Vertical Jump

***Effect Size***					***Forest Plots***
***Studies***	***Weight***	***Random, 95%CI***			***Random, 95% CI***
		***ES***	***Low***	***High***	
Gao (14) (Chinese Male)	15.15%	1.74	0.66	2.82	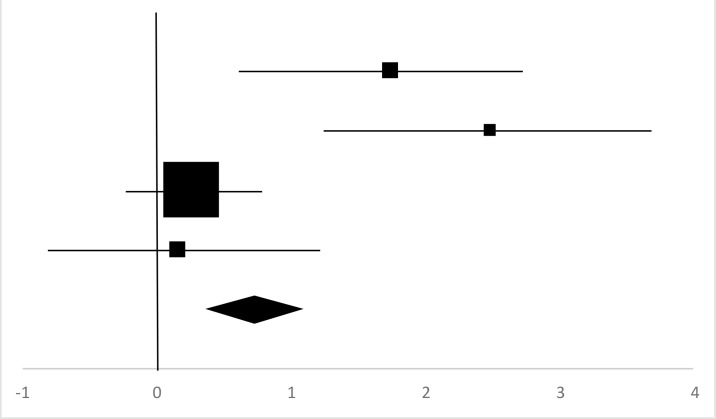
Gao (14) (Chinese Female)	12.08%	2.48	1.25	3.71	
Cuerdo & Pagaduan (17)	56.7%	0.26	−0.2	0.71	
Shi (19) (Chinese)	16.07%	0.15	−0.9	1.20	
Total	100%	0.73	0.29	1.18	Test for overall effect: Z=3.22 (*P*<0.01)

Heterogeneity: Q=15.81, *df*=4, C=15.81, T^2^=0.81

### Push-ups

Three sets of the Push-Up data from three studies involving 54 participants used in the meta-analysis (17, 19, 20). Overall, there was a significant difference between the pre-test and posttest in the Push-Up (95%CI, 0.35–1.32), and the mean EF is 0.84, *P*<0.01 in Table 9, considered a large ES.

**Table 9:** Effective size (ES) and forest plot of Push-Up

***Effect Size***					***Forest Plots***
***Studies***	***Weight***	***Random, 95%CI***			***Random, 95% CI***
		***ES***	***Low***	***High***	
Cuerdo & Pagaduan (17)	65.31%	0.50	0.03	0.96	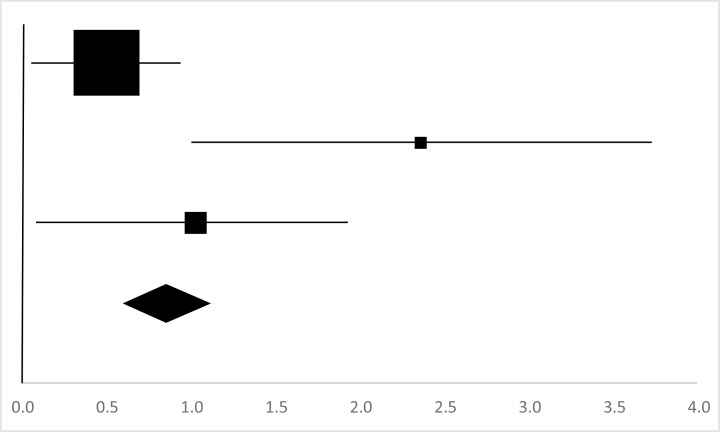
Shi (19)	11.64%	2.36	1.00	3.73	
(Chinese)					
Shi (20) (Chinese)	23.05%	1.03	0.09	1.96	
Total	100%	0.84	0.35	1.32	Test for overall effect: Z=3.39 (*P*<0.01)

Heterogeneity: Q=6.5, *df*=3, C=11.2, T^2^=0.4

### Pull-Ups

Four sets of the Pull-Ups data from four studies involving 86 participants were used in the meta-analysis (13, 17–19). Overall, there was a significant difference between the pre-test and posttest in the Pull-Ups (95% CI, 0.7–1.49), and the mean ES is 1.09, *P*<0.01 in Table 10, which is a very large ES.

**Table 10:** Effective size (ES) and forest plot of Pull-Ups

***Effect Size***					***Forest Plots***
***Studies***	***Weight***	***Random, 95%CI***			***Random, 95% CI***
		***ES***	***Low***	***High***	
Li (13) (Chinese)	16.51%	2.91	2.02	3.8	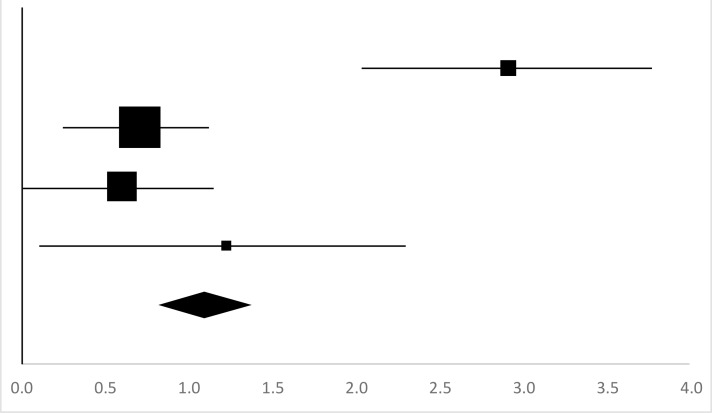
Cuerdo & Pagaduan (17)	42.43%	0.71	0.24	1.18	
Kasundra & Jethwa (18)	30.38%	0.6	0	1.2	
Shi (19) (Chinese)	10.67%	1.23	0.08	2.37	
Total	100%	1.09	0.7	1.49	Test for overall effect: Z=5.46 (*P*<0.01)

Heterogeneity: Q=20.92, *df*=4, C=24.38, T^2^=0.73

### Sit-Ups

Three sets of the sit-ups data from three studies involving 41 participants were used in the meta-analysis (18–20). Overall, there was a significant difference between the pre-test and posttest in the in the Sit-Ups (95%CI, 0.62–1.70), and the mean EF is 1.16, *p*<0.01 in Table 11, considered a large ES.

**Table 11:** Effective size (ES) and forest plot of Sit-Ups

***Effect Size***					***Forest Plots***
***Studies***	***Weight***	***Random, 95%CI***			***Random, 95% CI***
		***ES***	***Low***	***High***	
Kasundra & Jethwa (18)	50.86%	1.29	0.64	1.94	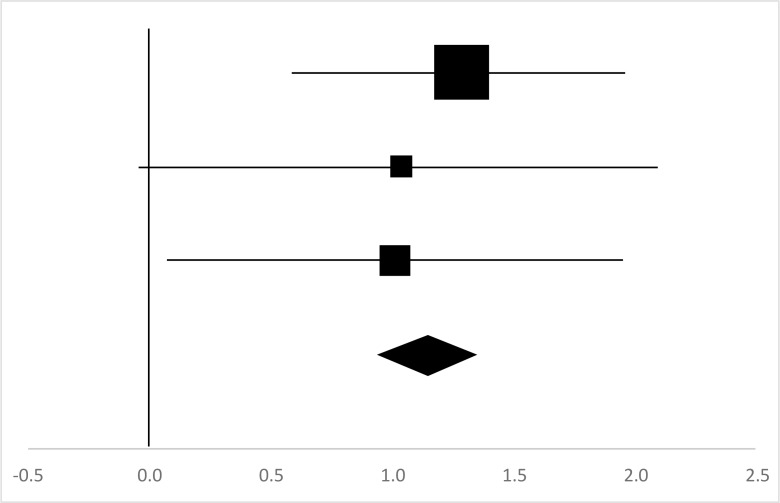
Shi (19) (Chinese)	20.69%	1.04	−0.08	2.16	
Shi (20) (Chinese)	28.45%	1.01	0.08	1.94	
Total	100%	1.16	0.62	1.70	Test for overall effect: Z=4.24 (*P* <0.01)

Heterogeneity: Q=0.38, *df*=3, C=10.77, T^2^=−0.15

### Sit-and-Reach

Four sets of Sit-and-Reach data from three studies involving 58 participants were used in the meta-analysis (13–15, 20). Overall, there was a significant difference between the pre-test and posttest in the Sit-and-Reach (95% CI, 1.01–1.99), and the mean ES is 1.5, *P*<0.01 in Table 12, which is a very large ES.

**Table 12:** Effective size (ES) and forest plot of Sit-and-reach

***Effect Size***					***Forest Plots***
***Studies***	***Weight***	***Random, 95%CI***			***Random, 95% CI***
		***ES***	***Low***	***High***	
Li (13) (Chinese)	23.01%	3.26	2.31	4.21	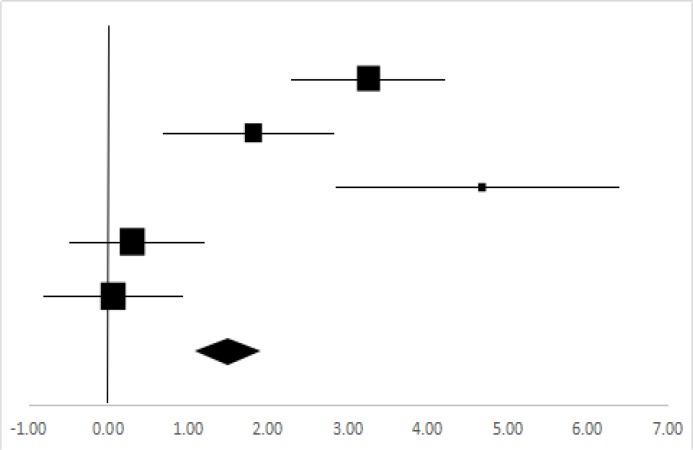
Gao (14) (Chinese Male)	17.7%	1.82	0.72	2.91	
Gao (14) (Chinese Female)	7.16%	4.68	2.89	6.47	
Jiang (15) (Chinese)	25.95%	0.3	−0.58	1.18	
Shi (20) (Chinese)	26.18%	0.06	−0.82	0.94	
Total	100%	1.5	1.01	1.99	Test for overall effect: Z=6.02 (*P*<0.01)

Heterogeneity: Q=43.66, *df*=5, C=15.54, T^2^=2.55

## Discussion

Considering the significance and the values of ES from the combined studies, results from the present meta-analysis demonstrated that rock climbing exercise would have significant effects on Handgrip strength, Lower limb pedaling power, Vertical jump, Push-ups, Pull-ups, Sit-ups and Sitand-reach (*P*<0.01), significant benefits on VO_2_max (*P*<0.05). However, rock climbing did not show significant effects on Body fat percentage and Heart rate.

### Body fat percentage

The Body Fat Percentage is an indicator of fitness level since it is the only body indicator in the present study which directly calculates a person’s relative body composition without regard to body height or weight (25). The results of the present study showed that, after rock climbing exercise, there was no significant difference in the Body Fat Percentage (ES=−0.83, *P*>0.05) in Table 3. This may be related to the length and frequency of experimental time, or may be also related to gender and activity level of the participants. When the intervention period is 24 wk and the body fat percentage of the participants was significantly decreased (14). However, when the intervention period is 4 wk, and Body Fat Percentage of the participants was not significantly decreased (17). If the intervention time was extended, Body Fat Percentage may be significantly reduced (16). The effect of rock climbing on body fat among college would remain for future study.

### Body function

Body function refers to the life activities demonstrated by the person as a whole and each organ system composing of it (26). VO_2_max and Heart Rate are the two indicators selected to reflect the impact of rock climbing on body function in college students. Rock climbing has a significant effect on college students’ VO_2_max (ES=0.76, *P*<0.05) in Table 4, but no significant effect on heart rate (ES= −0.79).

VO_2_max is the maximum rate of oxygen consumption as measured during incremental exercise. It reflects the aerobic physical fitness of the individual and is an important determinant of their endurance capacity during prolonged, sub-maximal exercise (27). In rock climbing, climbers need to overcome their own weight and move upward when they are climbing, which is equivalent to weight-bearing exercise. In addition, the coordination of the hands and feet on the rock contacting points may require extensive muscle forces so that rock climbing may result in increasing exercise intensity. During the climbing, climbers often stretch muscle fibers, increase the body’s blood circulation and metabolism, and increase muscle oxygen carrying capacity, so that the maximum oxygen uptake may be improved. As frequent stretching of the muscle fibers may increase micro blood circulation and metabolism which may improve muscle aerobic capacity, muscle strength and finally improve the VO_2_max (28).

Heart rate is the heartbeat measured by the number of contractions of the cardiac muscle per minute (bpm) (29). A long training can lead to reduced resting heart rate and increased the cardiac output, plus the changes in dominating the nervous tension and sensitivity of the heart serve as the causes of the lowered heart rate (12, 14, 29). Rock climbing may improve the myocardial contraction force of college students in the short term, thereby reducing the heart rate. However, due to the relatively short time practice, the impact of rock climbing on the heart rate may not be significantly improved (Table 5). The participants’ heart rate increased after the rock climbing training (15) and this report is consistent with other studies (12, 14, 29). Therefore, further study on the impact of rock climbing on heart rate would be warranted.

### Muscle power

Muscle power is the ability to generate a maximal force in a short time in any accelerating motion. Muscle power may be more important than muscle strength when performing some activities of daily living such as quickly rising from a chair, fast climbing stairs, or crossing a street (30,31).

The present study selected handgrip strength (ES=0.81) in Table 6, lower limb pedaling power (ES=0.36) in Table 7 and vertical jump (ES=0.73) in Table 8 to reflect the implement of rock climbing on muscle power and strength. The rock climbing may significantly improve the muscle power and strength of the college students. The skills of rock climbing with handwork and foot-work have a direct effect on the muscle power and strength development in the college students (14). Significant improvements in core strength and trunk mobility, as well as grip strength, have been found in sedentary young adults after 8 wk of indoor climbing (32). Moreover, during climbing with the small rock contact points, the climber has to exert a tremendous force to maintain the body balance and to change her/his positions during climbing. For the hand techniques, it is critical for the climber to fully use the power of the thumb, she/he tries to put the thumb on the back of fulcrum or cross ride of the index finger and middle finger so that the hand-grip force during climbing is increased. For the foot techniques, the climbers should manage their feet support and control the muscle strength and power. The present study selected vertical jump to reflect the explosive leg strength (33). Therefore, during the climbing, the pressing force from the lower limbs and vertical jump exercise is sustained and sufficient.

### Muscle endurance

Muscular endurance is the ability of a person to perform a long time continuous muscle work, namely the ability to fight fatigue (34). The present study selected push-ups (ES=0.84) in Table 9, and pull-ups (ES=1.09) in Table 10 and sit-ups (ES=1.16) in Table 11 as variables to reflect the impact of rock climbing on muscle endurance. Rock climbing has a significant effect on muscle endurance of the college students. Push-ups are an indicator reflecting the upper body strength endurance of extensor muscle group (35). Pull-ups are an indicator reflecting the endurance of upper body centripetal force from the flexor muscle group (35). Sit-ups are an indicator reflecting the endurance of the core muscle groups over fatigue within a given time (20).

During the climbing exercises, especially during the games or competitions, muscular endurance of the college students is greatly improved and promoted (36). In order for climbers to complete the climbing task successfully, they need to overcome muscle fatigue period to maintain good physical strength. Thus, after a period of rock climbing, muscular endurance of the college students can be significantly improved.

### Flexibility

Flexibility may be defined as the range of motion at a single joint or a series of joints (37). It is often mentioned in rock climbing instructional books that flexibility is an important component of the physical fitness for climbing (38). As a measure of overall flexibility (39), the sit-and-reach was employed, but we acknowledged its limitations for assessing overall climbing-specific flexibility. The results showed that the impact of flexibility (ES=1.5, *P*<0.01) in rock climbing on the college students is very significant in Table 12.

While climbing, the climbers try to make the ideal movement and the best posture as they can safely stay on the rock wall in accordance with the climbing challenge. In order to achieve this, they may need to stretch the body and extremities to the maximum possible extension during climbing (40). In some cases, such as in competition, they have to tolerate muscle fatigue to continue stretching the body, which is the effective way to improve their flexibility. In addition, during the warm-up and relaxation exercises before and after the rock climbing, the stretches of all body segments would also facilitate the body flexibility (41, 42). Therefore, the rock climbing may significantly improve the flexibility of the college students.

## Conclusion

Rock climbing may significantly improve VO_2_max, Handgrip Strength, Lower Limb Pedaling Power, Vertical Jump, Push-Ups, Pull-Ups, Sit-Ups and Sit-and-Reach for the college students. Longer term engaged in rock climbing may have better results for the college students’ physical fitness. However, the impacts of rock climbing on body fat percentage and heart rate remain to be further researched.

## Ethical considerations

Ethical issues (Including plagiarism, informed consent, misconduct, data fabrication and/or falsification, double publication and/or submission, redundancy, etc.) have been completely observed by the authors.
